# Supplementary material for: A modelling approach to estimate the prevalence of treatment-resistant schizophrenia in the United States
Source: PLoS One. 2020 Jun 4;15(6):e0234121. doi: 10.1371/journal.pone.0234121 (PMC7272089; doi:10.1371/journal.pone.0234121)
Supplement: S1 File — (ZIP) [file pone.0234121.s001.zip › S1_R-Code.docx]

# R-Code: “app.R“

# The Shiny application is an incidence-prevalence-mortality (IPM) model for Treatment Resistant Schizophrenia Prevalence

### read packages

if (!require(shiny)) {install.packages("shiny")}

if (!require(shinythemes)) {install.packages("shinythemes")}

if (!require(shinydashboard)) {install.packages("shinydashboard")}

if (!require(DT)) {install.packages("DT")}

library(shiny)

library(shinythemes)

library(shinydashboard)

library(DT)

### Source script: TRS Prevalence Model

source("trs_script.R")

# UserInterface ----------------------------------------------------------------------

ui <- dashboardPage(

# UI header ----------------------------------------------------------------------

dashboardHeader(title = "Moerup et al.",

titleWidth = 300

),

# UI sidebar ----------------------------------------------------------------------

dashboardSidebar(

width = 300,

collapsed = FALSE,

sidebarMenu(

#Schizophrenia

menuItem("Schizophrenia", tabName = "schizophrenia", startExpanded = TRUE,

menuSubItem("Treatment Resistant Schizophrenia", tabName = "treatmentresistantschizophrenia")

)

)

),

# UI body ----------------------------------------------------------------------

dashboardBody(

#Schizophrenia ----------------------------------------------------------------------

tabItem(tabName = "schizophrenia"

),

#Treatment Resistant Schizophrenia Page ----------------------------------------------------------------------

tabItem(tabName = "treatmentresistantschizophrenia",

fluidRow(

#Header

column(

width = 12,

h1("Prevalence of Treatment Resistant Schizophrenia"),

align="center",

h1(""),

fluidRow(

# left column

column(

width = 6,

# Enter SZ-incidence

box(

width = 12,

helpText("Enter an incidence rate for schizophrenia per 10.000", align = "center"),

sliderInput(inputId = "sz_men", label = "Men", min=0, max=15, value=1.50, step=0.01, animate = FALSE),

sliderInput(inputId = "sz_women", label = "Women", min=0, max=15, value=1.00, step=0.01, animate = FALSE)

),

# Select TRS Proxy Input

box(

width = 12,

helpText("Select Criteria for the incidence rate of Treatment Resistant Schizophrenia (TRS)",align="center"),

selectInput(inputId = "TRS", label = "Select TRS Proxy", choices = c("1 - Clozapine initiation","2 - Eligible for Clozapine","3 - Polypharmacy"), selected = "2 - Eligible for Clozapine")

),

# Select relative risk of Mortality

box(

width = 12,

helpText("Adjust the Relative Risk of Mortality to the general population for schizophrenia and TRS",align="center"),

box(

width = 6,

helpText("Schizophrenia",align="center"),

sliderInput(inputId = "sz_rr_men", label = "Men", min=0, max=10, value=2.80, step=0.01, animate=FALSE),

sliderInput(inputId = "sz_rr_women", label = "Women", min=0, max=10, value=2.50, step=0.01, animate=FALSE)

),

box(

width = 6,

helpText("Treatment-Resistant Schizophrenia", align = "center"),

sliderInput(inputId = "trs_rr_men", label = "Men", min=0, max=10, value=2.80, step=0.01, animate=FALSE),

sliderInput(inputId = "trs_rr_women", label = "Women", min=0, max=10, value=2.50, step=0.01, animate=FALSE)

)

),

# adjust start and end year

box(

width = 12,

sliderInput(inputId = "start_year", label = "Adjust the start year", min=1930, max=2014, value=1930, round=TRUE, ticks=FALSE, animate=FALSE, sep=""),

sliderInput(inputId = "end_year", label = "Adjust the end year", min=2014, max=2049, value=2014, round=TRUE, ticks=FALSE, animate=FALSE, sep="")

)

),

# right column

column(

width = 6,

# Output Table

box(

width = 12,

navbarPage(

h6("Table Output"),

#position = "fixed-bottom",

tabPanel(

h6("Prevalence per 10.000"),

width = 12,

DTOutput("trs_result"),

height = "auto"

)

)

)

)

)

)

)

)

)

)

# Server function ----------------------------------------------------------------------

server <- function(input, output) {

# Function for trs_result (prevalence) ----------------------------------------------------------------------

output$trs_result <- renderDT({

#bar indicating the program is calculating

progress <- shiny::Progress$new()

on.exit(progress$close())

progress$set(message = "Calculating...", value = 100)

incidence_male <- as.numeric(input$sz_men)

incidence_female <- as.numeric(input$sz_women)

TRS_proxy <- as.numeric(switch(input$TRS,

"1 - Clozapine initiation" = 1,

"2 - Eligible for Clozapine" = 2,

"3 - Polypharmacy" = 3))

SZ_RR_male <- as.numeric(input$sz_rr_men)

SZ_RR_female <- as.numeric(input$sz_rr_women)

TRS_RR_male <- as.numeric(input$trs_rr_men)

TRS_RR_female <- as.numeric(input$trs_rr_women)

start_year <- input$start_year

end_year <- input$end_year

calculate_prevalence(incidence_male, incidence_female, TRS_proxy,

SZ_RR_male, SZ_RR_female,

TRS_RR_male, TRS_RR_female,

start_year, end_year)

}

)

}

# Run the application ----------------------------------------------------------------------

shinyApp(ui = ui, server = server)

# R-Code: “trs_script.R“

########## read packages and import data ##########

if (!require(readxl)) {install.packages("readxl")}

if (!require(tidyverse)) {install.packages("tidyverse")}

if (!require(DT)) {install.packages("DT")}

library(readxl)

library(tidyverse)

library(DT)

lifetables_men_US = read_excel("data/trs/lifetables_men_US.xlsx")

lifetables_women_US = read_excel("data/trs/lifetables_women_US.xlsx")

population_US = read_excel("data/trs/population_US.xlsx")

incidence_thorup = read_excel("data/trs/incidence_thorup.xlsx")

TRS_age_distribution_US = read_excel("data/trs/TRS_age_distribution_US.xlsx")

TRS_proxy_incidence = read_excel("data/trs/TRS_proxy_incidence.xlsx")

########## Define initial input variables ##########

incidence_male = 1.500 #input: per 10.000

incidence_female = 1.000 #input: per 10.000

TRS_proxy = 2 #input: proxy criteria range "1-3"

SZ_RR_male = 2.800 #relative to general population

SZ_RR_female = 2.500 #relative to generla population

TRS_RR_male = 2.800 #relative to general population

TRS_RR_female = 2.500 #relative to general population

start_year = 1930 #first birth cohort year

end_year = 2014 #last birth cohort year

population_incidence = 28.9 #fixed input: per 100.000 (for thorup incidence weights)

########## Initial data manipulation ##########

# constructing sz incidence weights

incidence_weight_male = incidence_thorup$male / population_incidence #weights (distance from age specific and population incidence)

incidence_weight_female = incidence_thorup$female / population_incidence #weights (distance from age specific and population incidence)

incidence_thorup$incidence_weight_male = incidence_weight_male #inserting variable into data frame

incidence_thorup$incidence_weight_female = incidence_weight_female #inserting variable into data frame

# constructing TRS incidence probabilities based on inputs

TRS_incidence_US <- data.frame(age = c(13:75)) #construct a dataframe with a age variable

TRS_incidence_US$r_male_medicaid_c1 <- ifelse(TRS_incidence_US$age %in% c(13:17), TRS_proxy_incidence[1,2]*TRS_age_distribution_US[1,2],

ifelse(TRS_incidence_US$age %in% c(18:44), TRS_proxy_incidence[1,2]*TRS_age_distribution_US[2,2],

ifelse(TRS_incidence_US$age %in% c(45:64), TRS_proxy_incidence[1,2]*TRS_age_distribution_US[3,2],

ifelse(TRS_incidence_US$age %in% c(65:74), TRS_proxy_incidence[1,2]*TRS_age_distribution_US[4,2],

ifelse(TRS_incidence_US$age > 74, TRS_proxy_incidence[1,2]*TRS_age_distribution_US[5,2],0)))))

TRS_incidence_US$r_female_medicaid_c1 <- ifelse(TRS_incidence_US$age %in% c(13:17), TRS_proxy_incidence[1,2]*TRS_age_distribution_US[1,3],

ifelse(TRS_incidence_US$age %in% c(18:44), TRS_proxy_incidence[1,2]*TRS_age_distribution_US[2,3],

ifelse(TRS_incidence_US$age %in% c(45:64), TRS_proxy_incidence[1,2]*TRS_age_distribution_US[3,3],

ifelse(TRS_incidence_US$age %in% c(65:74), TRS_proxy_incidence[1,2]*TRS_age_distribution_US[4,3],

ifelse(TRS_incidence_US$age > 74, TRS_proxy_incidence[1,2]*TRS_age_distribution_US[5,3],0)))))

TRS_incidence_US$r_male_medicaid_c2 <- ifelse(TRS_incidence_US$age %in% c(13:17), TRS_proxy_incidence[2,2]*TRS_age_distribution_US[1,2],

ifelse(TRS_incidence_US$age %in% c(18:44), TRS_proxy_incidence[2,2]*TRS_age_distribution_US[2,2],

ifelse(TRS_incidence_US$age %in% c(45:64), TRS_proxy_incidence[2,2]*TRS_age_distribution_US[3,2],

ifelse(TRS_incidence_US$age %in% c(65:74), TRS_proxy_incidence[2,2]*TRS_age_distribution_US[4,2],

ifelse(TRS_incidence_US$age > 74, TRS_proxy_incidence[2,2]*TRS_age_distribution_US[5,2],0)))))

TRS_incidence_US$r_female_medicaid_c2 <- ifelse(TRS_incidence_US$age %in% c(13:17), TRS_proxy_incidence[2,2]*TRS_age_distribution_US[1,3],

ifelse(TRS_incidence_US$age %in% c(18:44), TRS_proxy_incidence[2,2]*TRS_age_distribution_US[2,3],

ifelse(TRS_incidence_US$age %in% c(45:64), TRS_proxy_incidence[2,2]*TRS_age_distribution_US[3,3],

ifelse(TRS_incidence_US$age %in% c(65:74), TRS_proxy_incidence[2,2]*TRS_age_distribution_US[4,3],

ifelse(TRS_incidence_US$age > 74, TRS_proxy_incidence[2,2]*TRS_age_distribution_US[5,3],0)))))

TRS_incidence_US$r_male_medicaid_c3 <- ifelse(TRS_incidence_US$age %in% c(13:17), TRS_proxy_incidence[3,2]*TRS_age_distribution_US[1,2],

ifelse(TRS_incidence_US$age %in% c(18:44), TRS_proxy_incidence[3,2]*TRS_age_distribution_US[2,2],

ifelse(TRS_incidence_US$age %in% c(45:64), TRS_proxy_incidence[3,2]*TRS_age_distribution_US[3,2],

ifelse(TRS_incidence_US$age %in% c(65:74), TRS_proxy_incidence[3,2]*TRS_age_distribution_US[4,2],

ifelse(TRS_incidence_US$age > 74, TRS_proxy_incidence[3,2]*TRS_age_distribution_US[5,2],0)))))

TRS_incidence_US$r_female_medicaid_c3 <- ifelse(TRS_incidence_US$age %in% c(13:17), TRS_proxy_incidence[3,2]*TRS_age_distribution_US[1,3],

ifelse(TRS_incidence_US$age %in% c(18:44), TRS_proxy_incidence[3,2]*TRS_age_distribution_US[2,3],

ifelse(TRS_incidence_US$age %in% c(45:64), TRS_proxy_incidence[3,2]*TRS_age_distribution_US[3,3],

ifelse(TRS_incidence_US$age %in% c(65:74), TRS_proxy_incidence[3,2]*TRS_age_distribution_US[4,3],

ifelse(TRS_incidence_US$age > 74, TRS_proxy_incidence[3,2]*TRS_age_distribution_US[5,3],0)))))

TRS_incidence_US$r_male_commercial_c1 <- ifelse(TRS_incidence_US$age %in% c(13:17), TRS_proxy_incidence[1,2]*TRS_age_distribution_US[1,4],

ifelse(TRS_incidence_US$age %in% c(18:44), TRS_proxy_incidence[1,2]*TRS_age_distribution_US[2,4],

ifelse(TRS_incidence_US$age %in% c(45:64), TRS_proxy_incidence[1,2]*TRS_age_distribution_US[3,4],

ifelse(TRS_incidence_US$age %in% c(65:74), TRS_proxy_incidence[1,2]*TRS_age_distribution_US[4,4],

ifelse(TRS_incidence_US$age > 74, TRS_proxy_incidence[1,2]*TRS_age_distribution_US[5,4],0)))))

TRS_incidence_US$r_female_commercial_c1 <- ifelse(TRS_incidence_US$age %in% c(13:17), TRS_proxy_incidence[1,2]*TRS_age_distribution_US[1,5],

ifelse(TRS_incidence_US$age %in% c(18:44), TRS_proxy_incidence[1,2]*TRS_age_distribution_US[2,5],

ifelse(TRS_incidence_US$age %in% c(45:64), TRS_proxy_incidence[1,2]*TRS_age_distribution_US[3,5],

ifelse(TRS_incidence_US$age %in% c(65:74), TRS_proxy_incidence[1,2]*TRS_age_distribution_US[4,5],

ifelse(TRS_incidence_US$age > 74, TRS_proxy_incidence[1,2]*TRS_age_distribution_US[5,5],0)))))

TRS_incidence_US$r_male_commercial_c2 <- ifelse(TRS_incidence_US$age %in% c(13:17), TRS_proxy_incidence[2,2]*TRS_age_distribution_US[1,4],

ifelse(TRS_incidence_US$age %in% c(18:44), TRS_proxy_incidence[2,2]*TRS_age_distribution_US[2,4],

ifelse(TRS_incidence_US$age %in% c(45:64), TRS_proxy_incidence[2,2]*TRS_age_distribution_US[3,4],

ifelse(TRS_incidence_US$age %in% c(65:74), TRS_proxy_incidence[2,2]*TRS_age_distribution_US[4,4],

ifelse(TRS_incidence_US$age > 74, TRS_proxy_incidence[2,2]*TRS_age_distribution_US[5,4],0)))))

TRS_incidence_US$r_female_commercial_c2 <- ifelse(TRS_incidence_US$age %in% c(13:17), TRS_proxy_incidence[2,2]*TRS_age_distribution_US[1,5],

ifelse(TRS_incidence_US$age %in% c(18:44), TRS_proxy_incidence[2,2]*TRS_age_distribution_US[2,5],

ifelse(TRS_incidence_US$age %in% c(45:64), TRS_proxy_incidence[2,2]*TRS_age_distribution_US[3,5],

ifelse(TRS_incidence_US$age %in% c(65:74), TRS_proxy_incidence[2,2]*TRS_age_distribution_US[4,5],

ifelse(TRS_incidence_US$age > 74, TRS_proxy_incidence[2,2]*TRS_age_distribution_US[5,5],0)))))

TRS_incidence_US$r_male_commercial_c3 <- ifelse(TRS_incidence_US$age %in% c(13:17), TRS_proxy_incidence[3,2]*TRS_age_distribution_US[1,4],

ifelse(TRS_incidence_US$age %in% c(18:44), TRS_proxy_incidence[3,2]*TRS_age_distribution_US[2,4],

ifelse(TRS_incidence_US$age %in% c(45:64), TRS_proxy_incidence[3,2]*TRS_age_distribution_US[3,4],

ifelse(TRS_incidence_US$age %in% c(65:74), TRS_proxy_incidence[3,2]*TRS_age_distribution_US[4,4],

ifelse(TRS_incidence_US$age > 74, TRS_proxy_incidence[3,2]*TRS_age_distribution_US[5,4],0)))))

TRS_incidence_US$r_female_commercial_c3 <- ifelse(TRS_incidence_US$age %in% c(13:17), TRS_proxy_incidence[3,2]*TRS_age_distribution_US[1,5],

ifelse(TRS_incidence_US$age %in% c(18:44), TRS_proxy_incidence[3,2]*TRS_age_distribution_US[2,5],

ifelse(TRS_incidence_US$age %in% c(45:64), TRS_proxy_incidence[3,2]*TRS_age_distribution_US[3,5],

ifelse(TRS_incidence_US$age %in% c(65:74), TRS_proxy_incidence[3,2]*TRS_age_distribution_US[4,5],

ifelse(TRS_incidence_US$age > 74, TRS_proxy_incidence[3,2]*TRS_age_distribution_US[5,5],0)))))

# Converting the rates to probabilities and inserting these into the data frame for TRS incidence

TRS_incidence_US$p_TRS_incidence_male_medicaid_c1 <- (1-exp(-((as.numeric(TRS_incidence_US$r_male_medicaid_c1)/100))))

TRS_incidence_US$p_TRS_incidence_female_medicaid_c1 <- (1-exp(-((as.numeric(TRS_incidence_US$r_female_medicaid_c1)/100))))

TRS_incidence_US$p_TRS_incidence_male_medicaid_c2 <- (1-exp(-((as.numeric(TRS_incidence_US$r_male_medicaid_c2)/100))))

TRS_incidence_US$p_TRS_incidence_female_medicaid_c2 <- (1-exp(-((as.numeric(TRS_incidence_US$r_female_medicaid_c2)/100))))

TRS_incidence_US$p_TRS_incidence_male_medicaid_c3 <- (1-exp(-((as.numeric(TRS_incidence_US$r_male_medicaid_c3)/100))))

TRS_incidence_US$p_TRS_incidence_female_medicaid_c3 <- (1-exp(-((as.numeric(TRS_incidence_US$r_female_medicaid_c3)/100))))

TRS_incidence_US$p_TRS_incidence_male_commercial_c1 <- (1-exp(-((as.numeric(TRS_incidence_US$r_male_commercial_c1)/100))))

TRS_incidence_US$p_TRS_incidence_female_commercial_c1 <- (1-exp(-((as.numeric(TRS_incidence_US$r_female_commercial_c1)/100))))

TRS_incidence_US$p_TRS_incidence_male_commercial_c2 <- (1-exp(-((as.numeric(TRS_incidence_US$r_male_commercial_c2)/100))))

TRS_incidence_US$p_TRS_incidence_female_commercial_c2 <- (1-exp(-((as.numeric(TRS_incidence_US$r_female_commercial_c2)/100))))

TRS_incidence_US$p_TRS_incidence_male_commercial_c3 <- (1-exp(-((as.numeric(TRS_incidence_US$r_male_commercial_c3)/100))))

TRS_incidence_US$p_TRS_incidence_female_commercial_c3 <- (1-exp(-((as.numeric(TRS_incidence_US$r_female_commercial_c3)/100))))

########## Calculate prevalence function ##########

calculate_prevalence <- function(incidence_male, incidence_female, TRS_proxy,

SZ_RR_male, SZ_RR_female,

TRS_RR_male, TRS_RR_female,

start_year, end_year){

########## Data Manipulation ##########

# adjusting incidence probabilities based on inputs

incidence_thorup$p_incidence_male = (1-exp(-(incidence_male*incidence_weight_male)/10000)) #converting rate to probability

incidence_thorup$p_incidence_female = (1-exp(-(incidence_female*incidence_weight_female)/10000)) #converting rate to probability

# Create data frame for final cohort distributions

markov_distribution = data.frame(year = c(start_year:end_year)) #Make dataframe to fit the start/end year

markov_distribution$Population_1 = NA #Add column for population

markov_distribution$Schizophrenia_1 = NA #add column for SZ

markov_distribution$TRS_1 = NA #add column for TRS

markov_distribution$Dead_1 = NA #Add column for Dead

markov_distribution$Population_2 = NA #Add column for population

markov_distribution$Schizophrenia_2 = NA #add column for SZ

markov_distribution$TRS_2 = NA #add column for TRS

markov_distribution$Dead_2 = NA #Add column for Dead

# Create Transition Matrix

rownames = c("Population", "Schizophrenia", "TRS", "Dead")

colnames = c("Population", "Schizophrenia", "TRS", "Dead")

transition_matrix = matrix(NA, nrow=4, ncol=4, byrow = TRUE, dimnames = list(rownames,colnames))

# Create matrix with initial distribution

initial_distribution = matrix(c(1,0,0,0), nrow=1, ncol=4, byrow = TRUE, dimnames = list("distribution",colnames))

# Store start_year variable to reset loop

start_year_reset <- start_year

########## 4. Cohort Simulation for Males ##########

# loop through genders (1 = male, 2 = female)

gender <- seq(1:2)

for (g in gender) {

if (g == 2){

start_year <- start_year_reset} # reset start_year for female

# Initial settings of variables before looping

calender_time = seq(start_year,end_year) #sequential variable for looping through calender time

num_year = c(0:(end_year-start_year)) # for later data manipulations with population/cohort

# loop through birth cohorts in calendertime (1930-2050)

for (start_year in calender_time) { #loop for 1930 til 2050

age = 0 #age variable for loops

age_endyear = end_year - start_year

# loop through age within each birth cohort

while (age <= age_endyear) { #loop for age until age 84 or year 2014

# Update transition probabilities conditional on age and year of birth

# pop to sz

if(age < 15) {

pop_sz = 0

} else if(age >= 15 & age <= 70 & g == 1) {

pop_sz = as.numeric(incidence_thorup[(age-14) , 6])

} else if(age >= 15 & age <= 70 & g == 2) {

pop_sz = as.numeric(incidence_thorup[(age-14) , 7])

} else if(age > 70) {

pop_sz = 0

}

# pop to TRS

pop_trs = 0

# pop to dead

pop_dead <- if(g == 1) {

as.numeric(lifetables_men_US[age + 1 , grep(start_year, colnames(lifetables_men_US))])

} else if(g == 2){

as.numeric(lifetables_women_US[age + 1 , grep(start_year, colnames(lifetables_women_US))])

}

# pop_pop

pop_pop = 1 - pop_sz - pop_dead - pop_trs

# sz_pop (no remission)

sz_pop = 0

# sz_TRS

if(age < 13) {

sz_trs = 0

} else if(age >= 13 & age <= 75 & g == 1) {

sz_trs = as.numeric(TRS_incidence_US[(age-12), ifelse(TRS_proxy == 1, 14, ifelse(TRS_proxy == 2, 16, ifelse(TRS_proxy == 3, 18,0)))])

} else if(age >= 13 & age <= 75 & g == 2) {

sz_trs = as.numeric(TRS_incidence_US[(age-12), ifelse(TRS_proxy == 1, 15, ifelse(TRS_proxy == 2, 17, ifelse(TRS_proxy == 3, 19,0)))])

} else if(age > 75) {

sz_trs = 0

}

# sz_dead

sz_dead = if(g == 1) {

pop_dead * SZ_RR_male

} else if(g == 2) {

pop_dead * SZ_RR_female

}

# sz_sz

sz_sz = 1 - sz_pop - sz_dead - sz_trs

# TRS_pop (remission)

trs_pop = 0

#TRS_SZ

trs_sz = 0

#trs_dead

trs_dead = if(g == 1) {

pop_dead * TRS_RR_male

} else if(g == 2) {

pop_dead * TRS_RR_female

}

#trs_trs

trs_trs = 1 - trs_pop - trs_sz - trs_dead

# dead_pop

dead_pop = 0

# dead to SZ

dead_sz = 0

# dead to trs

dead_trs = 0

# dead_dead

dead_dead = 1 - dead_pop - dead_sz - dead_trs

# Construct transition matrix based on updated variables

transition_matrix <- matrix(c( pop_pop , pop_sz , pop_trs , pop_dead ,

sz_pop , sz_sz , sz_trs , sz_dead ,

trs_pop , trs_sz , trs_trs , trs_dead ,

dead_pop , dead_sz , dead_trs , dead_dead ),

nrow=4, ncol=4, byrow = TRUE, dimnames = list(rownames,colnames))

# Multiply initial distribution with updated transition matrix

initial_distribution = (initial_distribution) %*% (transition_matrix)

# iterate age with 1

age = age + 1

} #age loop ends

# Add the final distribution to Markov_distribution data frame

markov_distribution[match(start_year,markov_distribution$year),(if(g == 1){2:5} else if(g == 2){6:9})] = initial_distribution

# Reset initial distribution for next birth cohort

initial_distribution = matrix(c(1,0,0,0), nrow=1, ncol=4, byrow = TRUE, dimnames = list("distribution",colnames))

# reset age variable for next birth cohort

age = 0

if (start_year == end_year) {break}

#iterate start_year to next birth cohort

start_year = start_year + 1

} #start_year loop ends

} #gender loop ends

########## 5. Adjust Markov distributions for dead ##########

markov_distribution$population_adj_1 <- NA

markov_distribution$Schizophrenia_adj_1 <- NA

markov_distribution$TRS_adj_1 <- NA

markov_distribution$Control_Column_1 <- NA

markov_distribution$population_adj_2 <- NA

markov_distribution$Schizophrenia_adj_2 <- NA

markov_distribution$TRS_adj_2 <- NA

markov_distribution$Control_Column_2 <- NA

for (g in gender){

for (i in num_year) {

markov_distribution[1+i,(if(g==1){10} else if(g==2){14})] <- as.numeric(markov_distribution[1+i,(if(g==1){2} else if(g==2){6})] / (rowSums(markov_distribution[1+i,(if(g==1){2:4} else if(g==2){6:8})], na.rm = FALSE, dims = 1)))

markov_distribution[1+i,(if(g==1){11} else if(g==2){15})] <- as.numeric(markov_distribution[1+i,(if(g==1){3} else if(g==2){7})] / (rowSums(markov_distribution[1+i,(if(g==1){2:4} else if(g==2){6:8})], na.rm = FALSE, dims = 1)))

markov_distribution[1+i,(if(g==1){12} else if(g==2){16})] <- as.numeric(markov_distribution[1+i,(if(g==1){4} else if(g==2){8})] / (rowSums(markov_distribution[1+i,(if(g==1){2:4} else if(g==2){6:8})], na.rm = FALSE, dims = 1)))

markov_distribution[1+i,(if(g==1){13} else if(g==2){17})] <- rowSums(markov_distribution[1+i,(if(g==1){10:12} else if(g==2){14:16})], na.rm = FALSE, dims = 1) # control column to check it sums to 1

}

}

########## 6. Multiply with population and calculate prevalence ##########

population_distribution <- data.frame(year = calender_time, age = rev(num_year),

Population_1 = NA, Schizophrenia_1 = NA, TRS_1 = NA, Total_1 = NA,

Population_2 = NA, Schizophrenia_2 = NA, TRS_2 = NA, Total_2 = NA,

Grand_Total = NA) # create dataframe for cohort results

for (g in gender) {

for (i in num_year) {

population_distribution[1+i,(if(g==1){3} else if(g==2){7})] <- round(markov_distribution[1+i,(if(g==1){10} else if(g==2){14})]*

population_US[length(num_year)-i,(if(g==1){2+((end_year-2014)*2)} else if(g==2){3+((end_year-2014)*2)})], digits = 0)

population_distribution[1+i,(if(g==1){4} else if(g==2){8})] <- round(markov_distribution[1+i,(if(g==1){11} else if(g==2){15})]*

population_US[length(num_year)-i,(if(g==1){2+((end_year-2014)*2)} else if(g==2){3+((end_year-2014)*2)})], digits = 0)

population_distribution[1+i,(if(g==1){5} else if(g==2){9})] <- round(markov_distribution[1+i,(if(g==1){12} else if(g==2){16})]*

population_US[length(num_year)-i,(if(g==1){2+((end_year-2014)*2)} else if(g==2){3+((end_year-2014)*2)})], digits = 0)

population_distribution[1+i,(if(g==1){6} else if(g==2){10})] <- rowSums(population_distribution[1+i,(if(g==1){3:5} else if(g==2){7:9})], na.rm = FALSE, dims = 1)

population_distribution[1+i,(if(g==2){11})] <- sum(population_distribution[1+i,6], na.rm = FALSE) + sum(population_distribution[1+i,10], na.rm = FALSE)

}

}

# add total row

rownames(population_distribution[length(num_year)+1,]) <- "Total"

population_distribution["Total",] <- c(1)

population_distribution["Total",] <- c(NA,

NA,

sum(population_distribution$Population_1),

sum(population_distribution$Schizophrenia_1),

sum(population_distribution$TRS_1),

sum(population_distribution$Total_1),

sum(population_distribution$Population_2),

sum(population_distribution$Schizophrenia_2),

sum(population_distribution$TRS_2),

sum(population_distribution$Total_2),

sum(population_distribution$Grand_Total))

# Create result data frame with prevalence calculations

result_df <- data.frame("Schizophrenia" = c(format(round((rowSums(population_distribution["Total",4:5], na.rm = FALSE, dims = 1)/(population_distribution["Total",6]/10000)),digits=1),nsmall=1),

format(round((rowSums(population_distribution["Total",8:9], na.rm = FALSE, dims = 1)/(population_distribution["Total",10]/10000)),digits=1),nsmall=1),

format(round(((rowSums(population_distribution["Total",4:5], na.rm = FALSE, dims = 1)+rowSums(population_distribution["Total",8:9], na.rm = FALSE, dims = 1))/

(population_distribution["Total",11]/10000)),digits=1),nsmall=1)

),

"Schizophrenia (only)" = c(format(round((sum(population_distribution["Total",4], na.rm = FALSE, dims = 1)/(population_distribution["Total",6]/10000)),digits=1),nsmall=1),

format(round((sum(population_distribution["Total",8], na.rm = FALSE, dims = 1)/(population_distribution["Total",10]/10000)),digits=1),nsmall=1),

format(round(((sum(population_distribution["Total",4], na.rm = FALSE, dims = 1)+sum(population_distribution["Total",8], na.rm = FALSE, dims = 1))/

(population_distribution["Total",11]/10000)),digits=1),nsmall=1)

),

"TRS" = c(format(round((sum(population_distribution["Total",5], na.rm = FALSE, dims = 1)/(population_distribution["Total",6]/10000)),digits=1),nsmall=1),

format(round((sum(population_distribution["Total",9], na.rm = FALSE, dims = 1)/(population_distribution["Total",10]/10000)),digits=1),nsmall=1),

format(round(((sum(population_distribution["Total",5], na.rm = FALSE, dims = 1)+sum(population_distribution["Total",9], na.rm = FALSE, dims = 1))/

(population_distribution["Total",11]/10000)),digits=1),nsmall=1)

),

"Share with TRS" = c(paste(format(round((population_distribution["Total",5]/rowSums(population_distribution["Total",4:5], na.rm = FALSE, dims = 1))*100,digits=1),nsmall=1),"%"),

paste(format(round((population_distribution["Total",9]/rowSums(population_distribution["Total",8:9], na.rm = FALSE, dims = 1))*100,digits=1),nsmall=1),"%"),

paste(format(round(((sum(population_distribution["Total",5]+sum(population_distribution["Total",9]))/

(rowSums(population_distribution["Total",4:5])+rowSums(population_distribution["Total",8:9]))))*100,digits=1),nsmall=1),"%")

),

row.names = c("Male","Female","Total")

)

# Modify dataframe with DT, to produce stylish data table

result <- datatable(result_df, rownames = TRUE,

options = list(dom = "t",

columnDefs = list(list(className = 'dt-center', targets = 0:4))),

colnames = c("Schizophrenia", "Schizophrenia (only)", "TRS", "%-Share with TRS")

)

return(result)

}
