# Supplementary material for: A modelling approach to estimate the prevalence of treatment-resistant schizophrenia in the United States
Source: PLoS One. 2020 Jun 4;15(6):e0234121. doi: 10.1371/journal.pone.0234121 (PMC7272089; doi:10.1371/journal.pone.0234121)
Supplement: Attachment — Submitted filename: renamed_81378.docx [file pone.0234121.s003.docx]

**Author response to reviewers’ comments:**

Reviewer #2: The authors have largely addressed the issues raised and the paper is improved for it. One final suggestion would be to add a limitation to the paper clearly acknowledging the potential issues with data accuracy and reliability in the datasets they have used.

*Author response: We would like to thank the reviewer for the thoughtful comments on this and the previous version of the manuscript.

We added a paragraph in the limitation section focusing on potential issues with the data used to model TRS.*

Reviewer #4: Thank you for addressing my concerns, which are generally clear and have helped to improve the manuscript. One concern that I feels requires further detail and revision is over validation. The abstract of the paper states that the model was validated for the purpose of estimate the prevalence of TRS in the US. The author response to my earlier concern with this issue, however, noted that validity had been established by asking clinicians and statistical experts about the model and its methodological framework. This is not really internal validity but some measure of apparent or face validity. Internal validity would be more formally established using bootstrapping or some other cross-validation procedure applied to the available data. There is also no mention of any external validation (do the predictions from your model accurately predict TRS prevalence in the US in a representative sample?), which may not be possible in the absence of a reliable external dataset in which to test this. If so, external validation can simply be acknowledged as a limitation, but I would think there is more the authors could do to internally validate their models.

*Author response: We thank the reviewer for these thoughtful comments and for helping us improve the quality of the paper.*

*The reviewer raises an important point about the validation of the model. We deleted the statement in the abstract regarding validation, as the model, as noted by the reviewer, has not been thoroughly validated. We did expand the limitation section to address issues concerning external validation.

We do however disagree with the reviewer that we can do more regarding internal validity. The reviewer suggested more could be done using on internally validating the model by use of bootstrapping or cross-validation. Such approaches are very useful when data allows sampling, however the data used in the present simulation does not allow for such sampling. The life tables used to derive the* *age, gender and cohort specific mortality risks does not have any variation in it, nor does the population data. Regarding the epidemiological inputs, the base scenario for each proxy were based on the medians from published literature and were presented along with the 10^th^ and 90^th^ percentiles, which produced a deterministic range for the results.*
